# Temporal and Spatial Effects of Blast Overpressure on Blood-Brain Barrier Permeability in Traumatic Brain Injury

**DOI:** 10.1038/s41598-018-26813-7

**Published:** 2018-06-06

**Authors:** Matthew Kuriakose, Kakulavarapu V. Rama Rao, Daniel Younger, Namas Chandra

**Affiliations:** Center for Injury Biomechanics, Materials and Medicine (CIBM3), Department of Biomedical Engineering, New Jersey Institute of Technology, Newark, NJ 07102-1982 USA

## Abstract

Blast-induced traumatic brain injury (bTBI) is a “signature wound” in soldiers during training and in combat and has also become a major cause of morbidity in civilians due to increased insurgency. This work examines the role of blood-brain barrier (BBB) disruption as a result of both primary biomechanical and secondary biochemical injury mechanisms in bTBI. Extravasation of sodium fluorescein (NaF) and Evans blue (EB) tracers were used to demonstrate that compromise of the BBB occurs immediately following shock loading, increases in intensity up to 4 hours and returns back to normal in 24 hours. This BBB compromise occurs in multiple regions of the brain in the anterior-posterior direction of the shock wave, with maximum extravasation seen in the frontal cortex. Compromise of the BBB is confirmed by (a) extravasation of tracers into the brain, (b) quantification of tight-junction proteins (TJPs) in the brain and the blood, and (c) tracking specific blood-borne molecules into the brain and brain-specific proteins into the blood. Taken together, this work demonstrates that the BBB compromise occurs as a part of initial biomechanical loading and is a function of increasing blast overpressures.

## Introduction

Blast-induced traumatic brain injuries (bTBIs) are the signature wounds in military and civilian populations due to the increased use of improvised explosive devices and asymmetric warfare in military conflicts and acts of terrorism, domestically and overseas^1–3^. Despite the increase in studies related to bTBI in recent years, there is only a limited understanding of how blast waves interact with the brain and cause injury, which has precluded the establishment of comprehensive diagnostic criteria for bTBI and the potential of therapeutic strategies. A recent survey reported that more than 30 phase III clinical trials aimed at targeting TBI have failed^4–7^. Understanding how blast-induced neurotrauma displays a temporal and spatial evolution of neuropathology in various regions of the brain is critical for the identification of injury mechanisms and the development of preventative measures and treatments for bTBI patients. Among many mechanisms of injury, damage to the BBB has been identified as a potential candidate and has been the focus of several clinical and experimental investigations aimed to establish injury baselines and to determine the timelines for therapeutic interventions for neurotrauma^8,9^.

The blood-brain barrier (BBB) is a highly, selectively-permeable membrane that separates the brain from the circulatory system. The BBB is dynamically modulated by cellular interactions between endothelial cells, the tight junctions that join them, pericytes, and astrocytes that support the endothelial capillaries^10–16^. Many neurological disorders including stroke^17–20^, Alzheimer’s disease^21,22^, Parkinson’s disease^14,23^, HIV-1 encephalitis^16,24^, epilepsy^25,26^, and multiple sclerosis^27–29^ display impaired BBB permeability. BBB disruption is also one of the most frequently investigated mechanisms of injury in blunt TBI and has been commonly used to evaluate the degree and extent of injury^30–34^. Several groups have reported abnormal opening of the BBB in closed cortical injuries^35–37^, weight drop models^38,39^, and blast models^34,40–45^. Reported results are derived from different probing methodologies, with different injury models, at different injury intensities, in different spatial regions of the brain. A limited number of studies has implicated increased BBB permeability in the pathology of blast-induced traumatic brain injury but, to the author’s knowledge, no such investigation has resolved the temporal and spatial resolution of BBB changes in bTBI, especially as a function of increasing biomechanical blast loadings. While a number of groups have assessed the BBB permeability following blast^42,46–49^, it is important to note that not all blast models impart the same type of injury on experimental animals and, for this reason, authors compared the results of this work only to models that feature pure, primary blast injury void of secondary and tertiary effects^50,51^.

In this study, rats were exposed to a range of shock waves in a field-validated shock tube and permeability of the BBB was assayed by extravasation of Evans blue (which binds to albumin, a 66 kDa protein abundant in blood) and sodium fluorescein (a 376 Da molecule) in the frontal cortex, striatum, somatosensory barrel-field cortex, hippocampus, thalamus, and cerebellum. Rats were exposed to sub-mild (35 kPa), mild (70 kPa), mild-moderate (130 kPa), and moderate (180 kPa) blast overpressures; these classifications were based on a 24-hour survival dose-response of rodent models based on our previous results (see Fig. 2 of ref.^52^). Also, the Department of Defense (DOD) Instruction 6490.11 dated September 18, 2012 has established policies, responsibilities and procedures for mTBI occurring in the battlefield. When service members are involved in a potential concussive event, they are separated for medical observations and mandatory rest period. DOD defines potential concussive events as the presence of service members within 50 m of a blast or exposure to more than a single blast in a year^1^. Based on theoretical analysis, the peak blast overpressure of 180 kPa can occur at a distance of about 10 m for 100 kg TNT explosive. The same blast will produce 130 kPa BOP in about 12 m, 70 kPa in a distance of 16 m and 35 kPa in a distance of 23 m^53^. We had achieved these field blast loadings using operating characteristics like membrane thickness, transition length, driver gas, driver volume, and end plates^54^. Additionally, three different time-points post-injury (15 min, 4 hr, and 24 hr) were chosen in order to develop a temporal profile of BBB opening and to identify the extent of barrier breach.

## Results

### Primary blast induces breakdown of the blood-brain barrier

**Figure 1:**
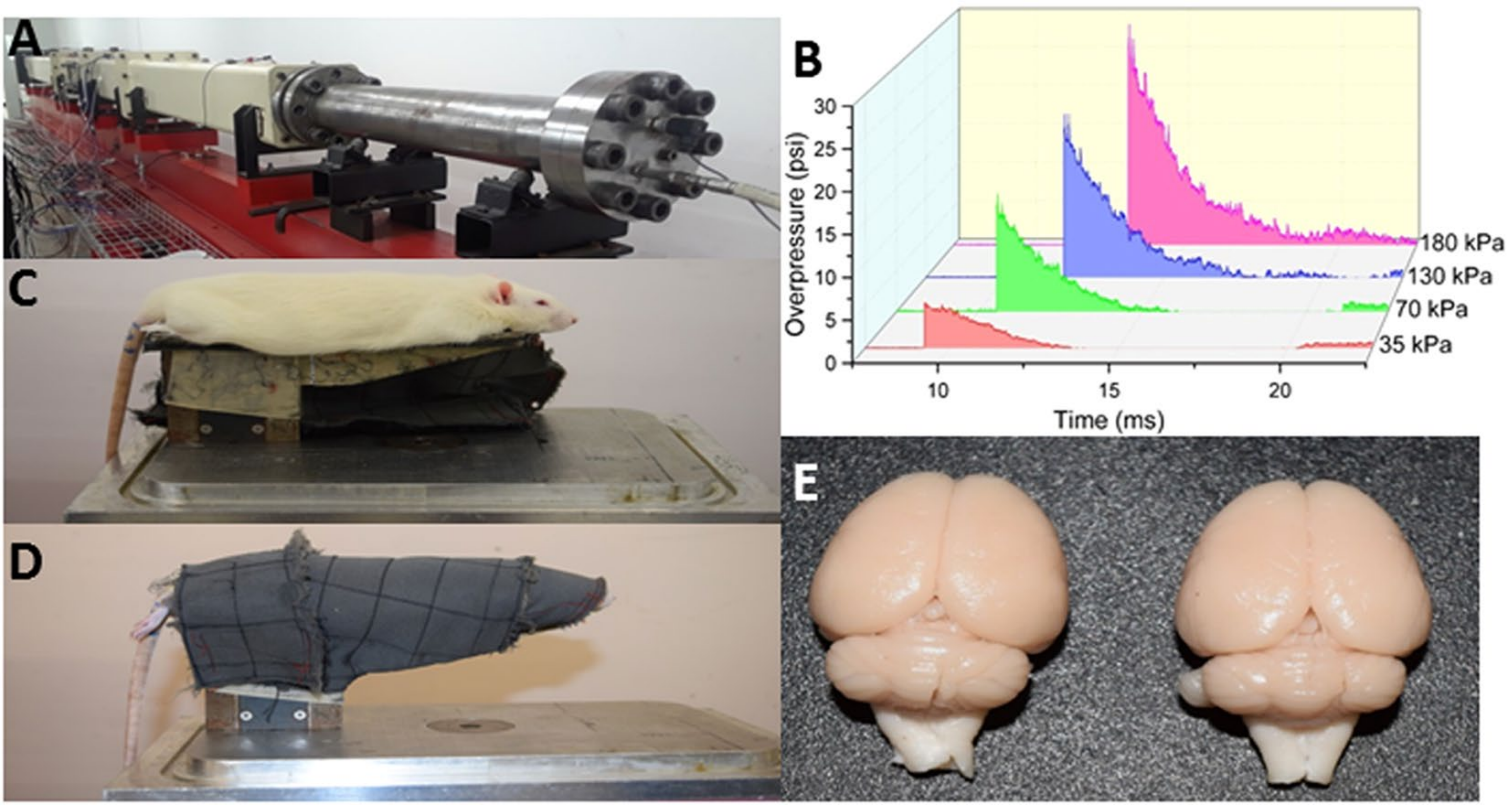
The shock tube housed in the blast laboratory in the Center for Injury Biomechanics, Materials, and Medicine at NJIT. (**A**) The 9-inch, square cross-section, 6 meters long shock tube instrumented with pressure sensors along the top of the shock tube. (**B**) Representative pressure-time profiles acquired from pressure sensors in the shock tube at the four overpressures used in this study. (**C**) Rat holder mounted in the test section of the shock tube, with rat placed in the prone position (top) and (**D**) tightly wrapped in a harness to minimize head and body motion during the blast (bottom). (**E**) Control (left) and injured (right) brains following perfusion-fixation. All blood in the neurovasculature has been washed away, as seen from the white appearance of brains, confirming that all tracers measured has leaked from the vessels into the brain parenchyma.

**Figure 2:**
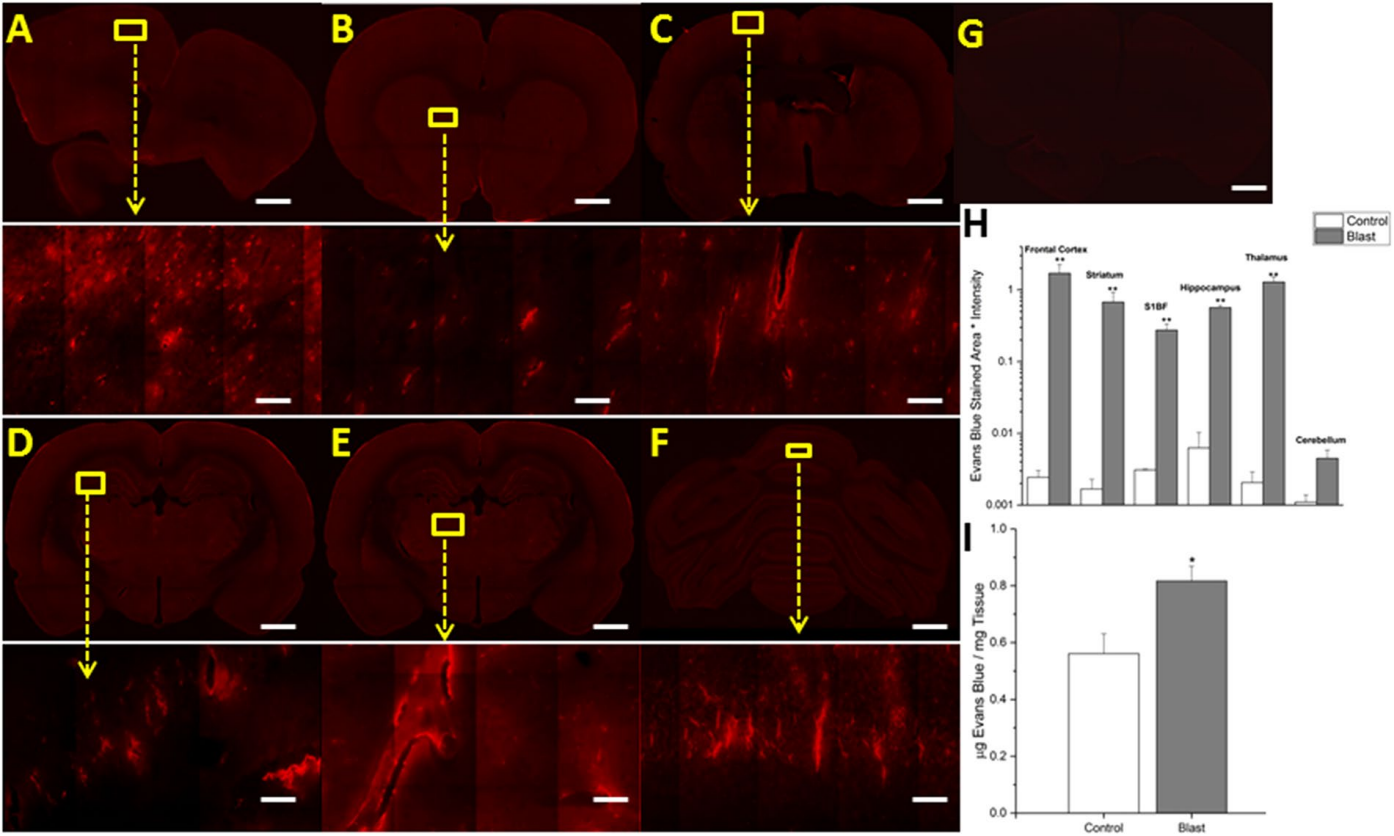
Fluorescent images of Evans blue extravasation. Images show 10x macro-shots as well as zoomed in 40x images in representative regions in the frontal cortex (**A**), striatum (**B**), somatosensory barrel-field cortex (**C**), hippocampus (**D**), thalamus (**E**) and cerebellum (**F**), 15 minutes following 180 kPa blast exposure. Control images were dramatically enhanced, yet still show limited visibility, due to the absence of extravasated dye. Frontal cortex was taken as a representative control image (**G**). Quantitation of extravasation is shown using a semilog plot in order to capture magnitudinal differences (**H**). Absorption spectrophotometry results of Evans blue in control and injured rats (n = 5) (**I**). *Indicates a difference in intensity compared with control with a statistical significance of p < 0.05, **Indicates p < 0.01. Scale bar = 1 mm in coronal sections and 50 μm in 40x images.

**Figure 3:**
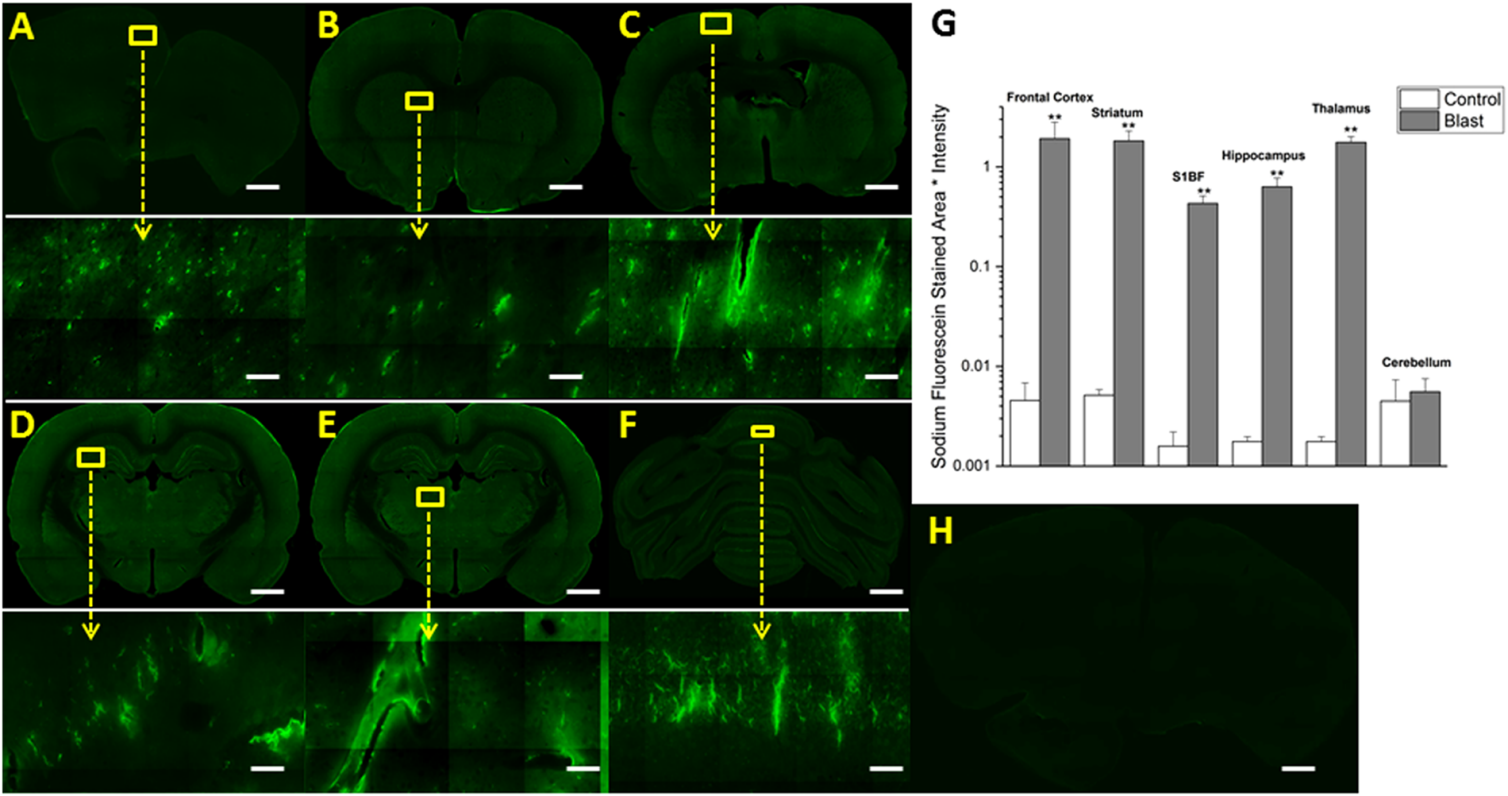
Fluorescent images of sodium fluorescein extravasation. Images show 10x macro-shots as well as zoomed in 40x images in representative regions in the frontal cortex (**A**), striatum (**B**), somatosensory barrel-field cortex (**C**), hippocampus (**D**), thalamus (**E**) and cerebellum (**F**) 15 minutes following 180 kPa blast exposure. Quantitation of extravasation is shown using a semilog plot in order to capture magnitudinal differences (**G**). Control images were dramatically enhanced, yet still show limited visibility, due to the absence of extravasated dye. Frontal cortex was taken as a representative image (**H**). **Indicates a difference in intensity compared with control with a statistical significance of p < 0.01. Scale bar = 1 mm in coronal sections and 50 μm in 40x images.

The blast injury model developed at NJIT, capable of reproducing field-relevant blast overpressures and previously characterized^52,55–57^ was used throughout this study (Fig. 1A,B). All test animals were mounted in the custom-made rat holder and placed in the test section of the shock tube (Fig. 1C). Rats were immobilized with the head restrained in order to ensure no confounding head movements or possible acceleration/deceleration that can be artifacts of this study (Fig. 1C,D). Rats were exposed to a single blast exposure of varying overpressures (see above) and subjected to transcardial perfusion (with phosphate buffered saline)-fixation (with 4% paraformaldehyde) within 15 min from exposure of animals to blast loading, which was the earliest time point that was assessed for BBB extravasation. The extent of extravasation of both sodium fluorescein and Evans blue was evaluated in all selected regions of the brain (Figs 2 and 3). These quantitative results support the hypothesis that blast-induced BBB opening allows for extravasation of molecules of 69 kDa or smaller immediately following injury (biomechanical loading) and, given an increase in molecular mass, extravasation of tracers was less widespread.

The fluorescence detection method of extravasation was also validated with absorption spectrophotometry for Evans blue. Animals (n = 6) exposed to 180 kPa shock wave showed an average of 45.6% increase in the tissue content of EB than control animals wherein the precise concentration of EB present in tissue was derived from a standard curve made of seven different EB dilutions (Fig. 2I).

### Different brain regions express different degrees of BBB permeability following moderate blast injury

After exposing animals to moderate blast (180 kPa), differential damage was observed in six different regions immediately following trauma (~15 min). In almost every region studied, statistically significant differences (p < 0.01), as determined by ANOVA followed by Tukey test, in the levels of both extravasated dyes was observed, highlighting the diffuse nature of bTBI (Figs 2 and 3). The quantitative values of the fluorescence intensities of EB (as measured by intensity x stained area) between control and blast injury groups in different brain regions are as follows: frontal cortex (control 0.002432, blast 1.699085, 700-fold, p = 0.006), striatum (control 0.001674, blast 0.675456, 400-fold, p = 0.001), somatosensory-barrel field cortex (control 0.003094, blast 0.274115, 90-fold, p = 0.009), hippocampus (control 0.006258, blast 0.564796, 90-fold, p = 0.001), thalamus (control 0.002056, blast 1.282525, 600-fold, p = 0.002) and cerebellum (control 0.001102, blast 0.00448, 4-fold, p > 0.05); and for sodium fluorescein: frontal cortex (control 0.004555, blast 1.91963, 400-fold, p = 0.008), striatum (control 0.005134, blast 1.822249, 300-fold, p = 0.008), somatosensory barrel-field cortex (control 0.001584, blast 0.429447, 250-fold, p = 0.009), hippocampus (control 0.00794, blast 0.630788, 80-fold, p = 0.009), thalamus (control 0.003556, blast 1.767197, 500-fold, p = 0.004) and cerebellum (control 0.004479, blast 0.055629, 30-fold, p > 0.05). The most robust changes occurred in the frontal cortex, striatum, and thalamus for both tracers while minimal to no statistically significant extravasation was observed in the cerebellum, which aligns well with results from previous investigations^34,58^. In every other region analyzed, there is at least a tenfold difference in the amount of extravasated dyes compared to controls in the acute time period.

### BBB permeability varies as a function of time following moderate blast injury

**Figure 4:**
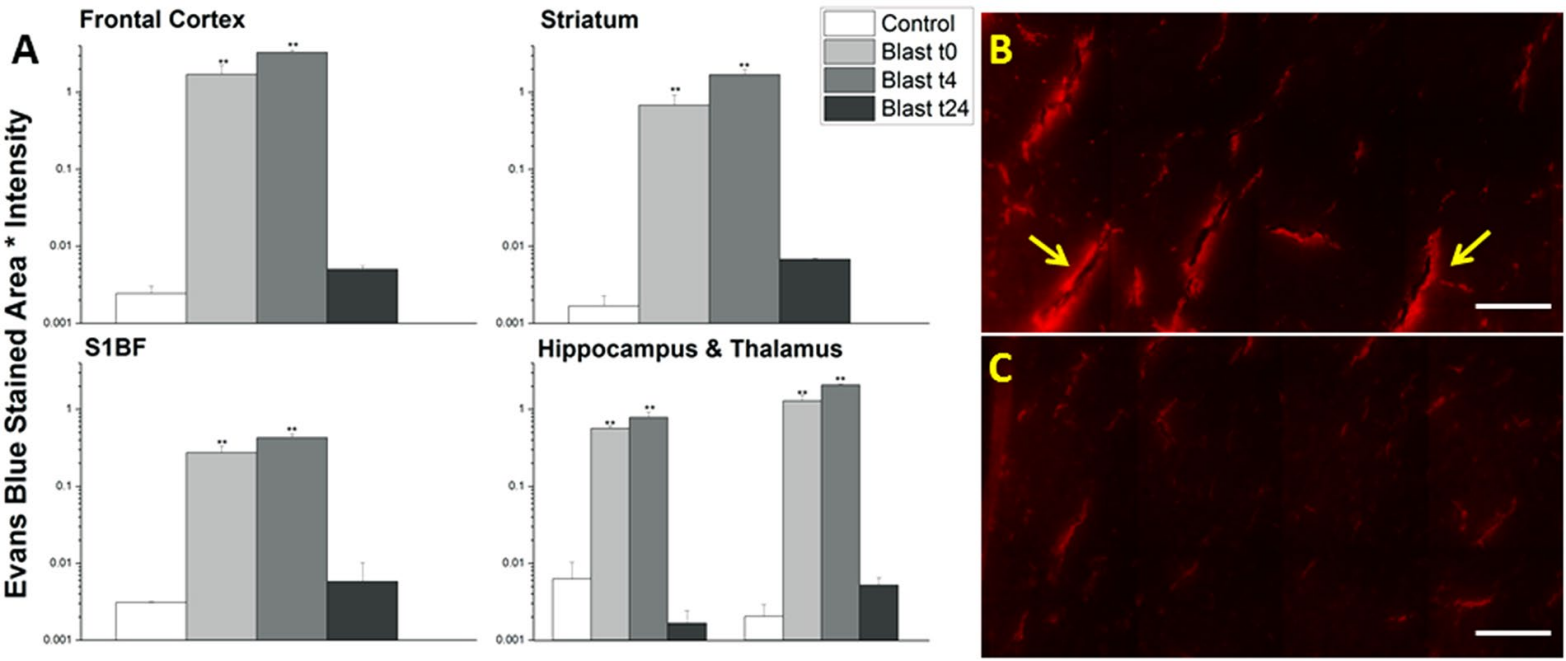
Quantitation of extravasation of Evans blue for 15 minutes (t0), 4 (t4), and 24 (t24) hours post-180 kPa blast exposure in frontal cortex, striatum, somatosensory barrel-field cortex, hippocampus, and thalamus using a semilog plot in order to capture magnitudinal differences (**A**). The frontal cortex was chosen for illustrative purposes and to qualitatively depict the difference between 4 (**B**) and 24 hrs (**C**). Arrows indicate areas of leakage from the vessels, which are more pronounced in 4 hours than any other time point studied in this investigation. **Indicates a difference in intensity compared with control with a statistical significance of p < 0.01. Scale bar = 100 μm.

**Figure 5:**
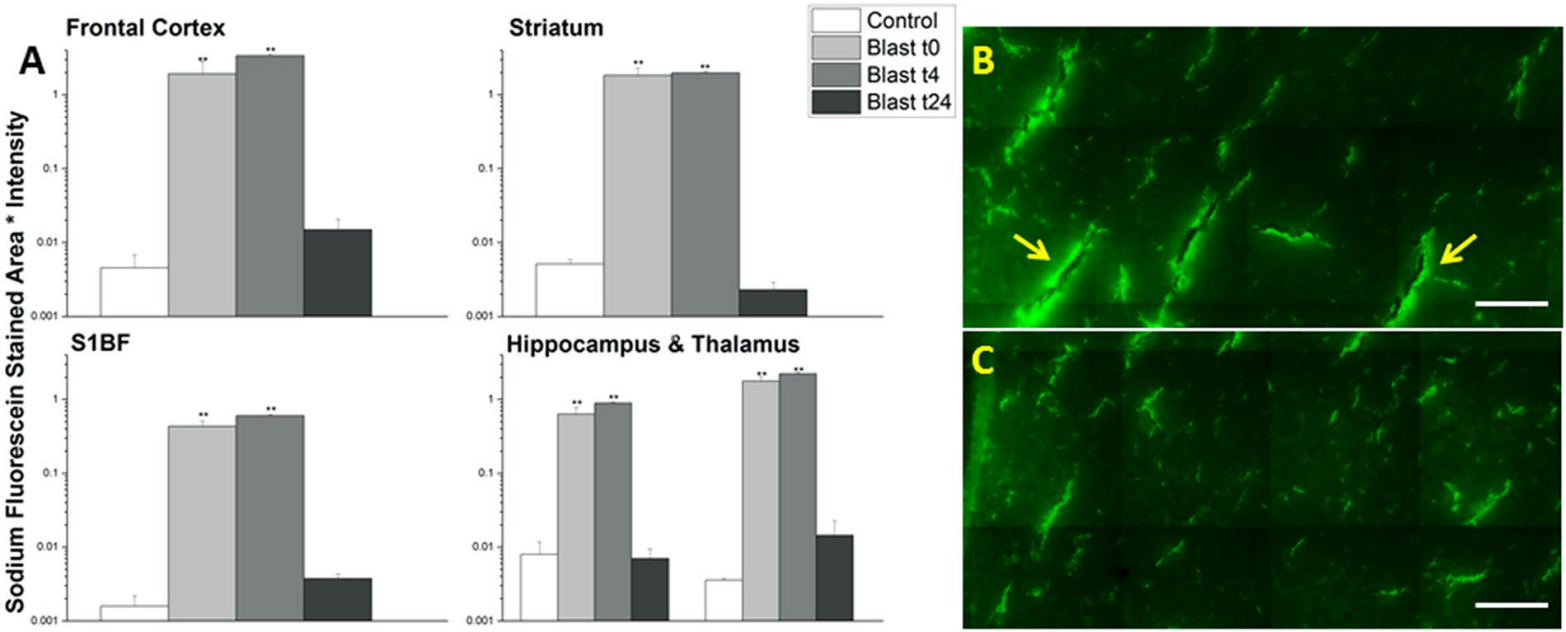
Quantitation of extravasation of sodium fluorescein for 15 minutes (t0), 4 (t4), and 24 (t24) hours post- 180 kPa blast exposure in frontal cortex, striatum, somatosensory barrel-field cortex, hippocampus, and thalamus using a semilog plot in order to capture magnitudinal differences (**A**). The frontal cortex was chosen for illustrative purposes and to qualitatively depict the difference between 4 **(B**) and 24 hrs (**C**). Arrows indicate areas of leakage from the vessels, which are more pronounced in 4 hours than any other time point studied in this investigation. **indicates a difference in intensity compared with control with a statistical significance of p < 0.01. Scale bar = 100 μm.

In order to determine the time-course for blood-brain barrier permeability following blast, groups of (n = 4–6 for each time point) rats were sacrificed at specified times post-injury (15 min, 4 hours, 24 hours). While the amount of extravasation was significant for both sodium fluorescein and Evans blue immediately after blast, there was an even greater increase in tracer penetration four hours following the blast exposure (Figs 4 and 5). Increases over controls were as follows for Evans blue at 4 hours, respectively (four hour blast values followed by fold-increase and 24 hour blast values followed by fold increase): frontal cortex (3.270751, 1300-fold, 0.005012, 2-fold), striatum (1.681731, 1000-fold, 0.006758, 4-fold), somatosensory barrel-field cortex (0.427674, 150-fold, 0.005808, 1-fold), hippocampus (0.781473, 120-fold, 0.00166, <1-fold), and thalamus (2.080952, 1000-fold, 0.005169, 2-fold) and for sodium fluorescein: frontal cortex (3.365124, 700-fold, 0.014903, 3-fold), striatum (1.966033, 400-fold, 0.00229, <1-fold), somatosensory barrel-field cortex (0.596185, 400-fold, 0.003732, 2-fold), hippocampus (0.885597, 100-fold, 0.006966, <1-fold), and thalamus (2.223883, 600-fold, 0.014472, 4-fold). Interestingly, 24 hours post-injury, the extravasation of EB and NaF returned back to that of control levels (Figs 4 and 5) suggesting possible resealing occurring at or before 24 hours.

**Figure 6:**
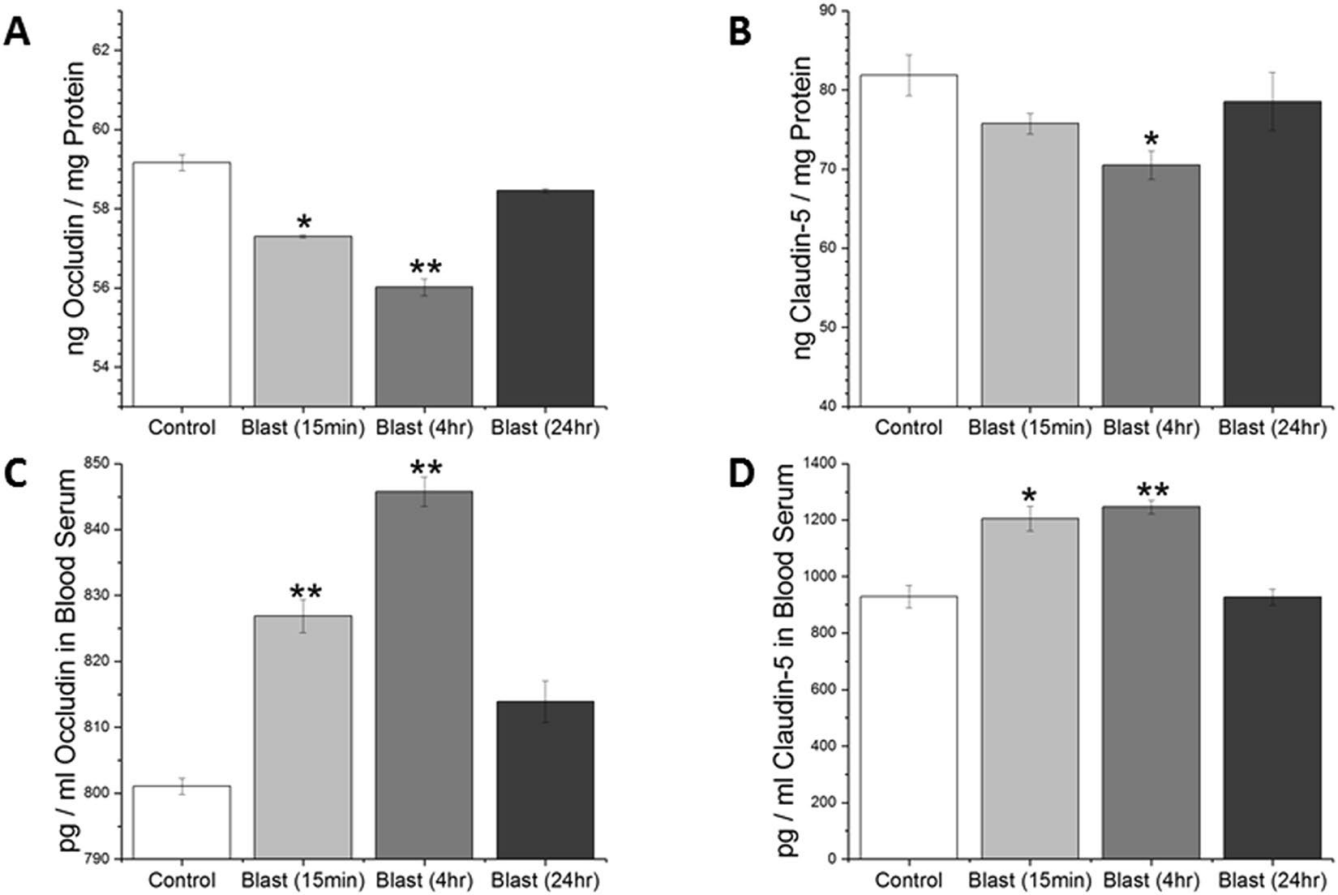
ELISA results for tight junction proteins occludin and claudin-5, respectively in brain (**A**,**B**) and blood serum (**C**,**D**). Assay conducted for blast (180 kPa BOP) samples fifteen minutes, four, and twenty four hours post-exposure and compared with controls. *Indicates a difference in intensity compared with control with a statistical significance of p < 0.05, **Indicates p < 0.01.

As an alternate means to investigate BBB disruption as well as to examine possible mechanisms of BBB permeability change following blast, levels of tight junction proteins (TJPs) occludin and claudin-5 were determined in lysates from cerebral hemispheres by quantitative ELISAs. Statistically significant reductions in tight junction protein abundance were observed at this time point, which serves as further evidence of the compromised BBB (Fig. 6). Noteworthy that such reduction in TJPs in brain lysates is accompanied by a concomitant increase in their levels in serum samples obtained from the same animals that are used for evaluation of brain levels of tight junction proteins. These data not only strongly suggest that shockwave propagated from blast is able to dislodge TJPs from the cerebral vasculature but also that these proteins translocate to blood.

### The extent of BBB permeability displays a tendency to increase as a function of blast overpressure

**Figure 7:**
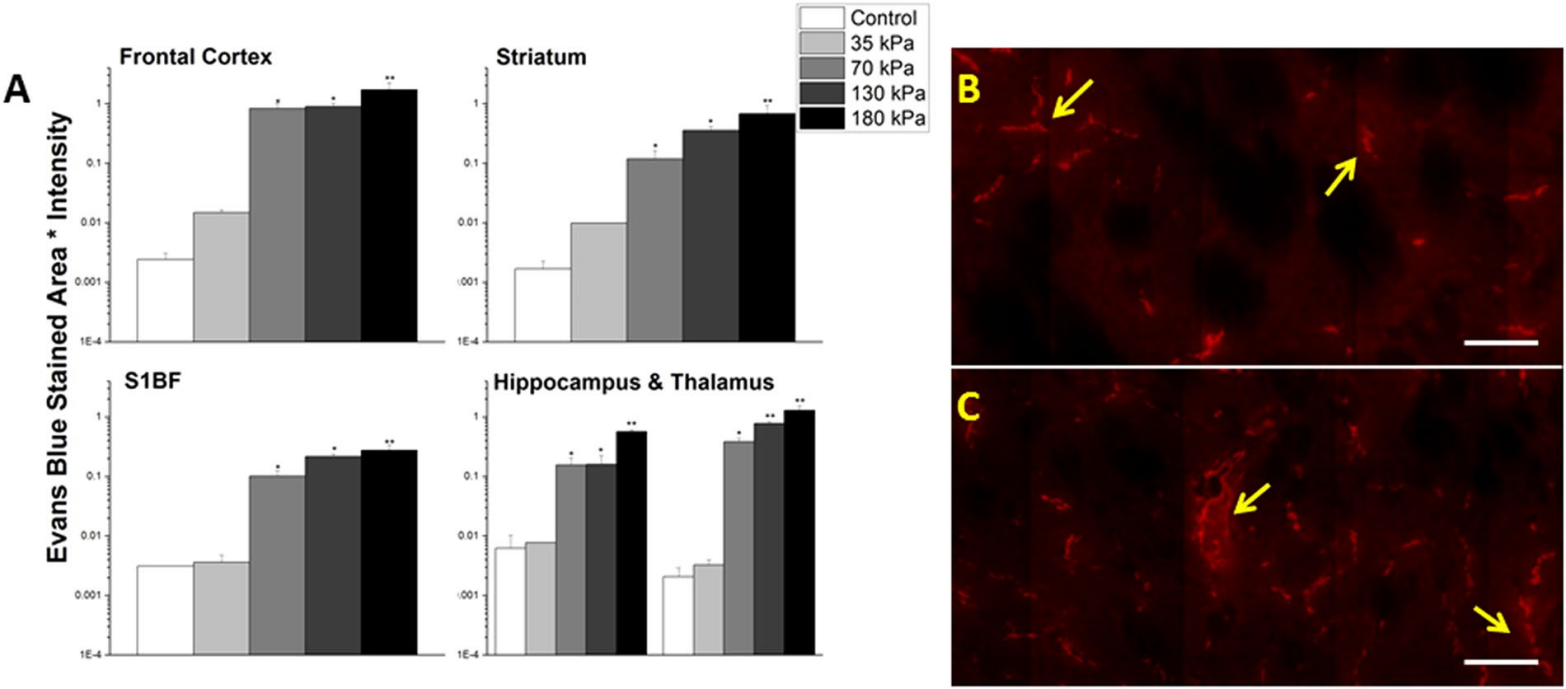
Quantitation of extravasation of Evans blue for 35, 70, 130, and 180 kPa blast overpressures, 15 minutes post-exposure in frontal cortex, striatum, somatosensory barrel-field cortex, hippocampus, and thalamus using a semilog plot in order to capture magnitudinal differences (**A**). The striatum was chosen for illustrative purposes and to qualitatively depict the difference between 70 (**B**) and 130 kPa (**C**). Leaks appear longer and more intense with increasing overpressure in the same brain regions. *Indicates a difference in intensity compared with control with a statistical significance of p < 0.05, **Indicates p < 0.01. Scale bar = 100 μm.

**Figure 8:**
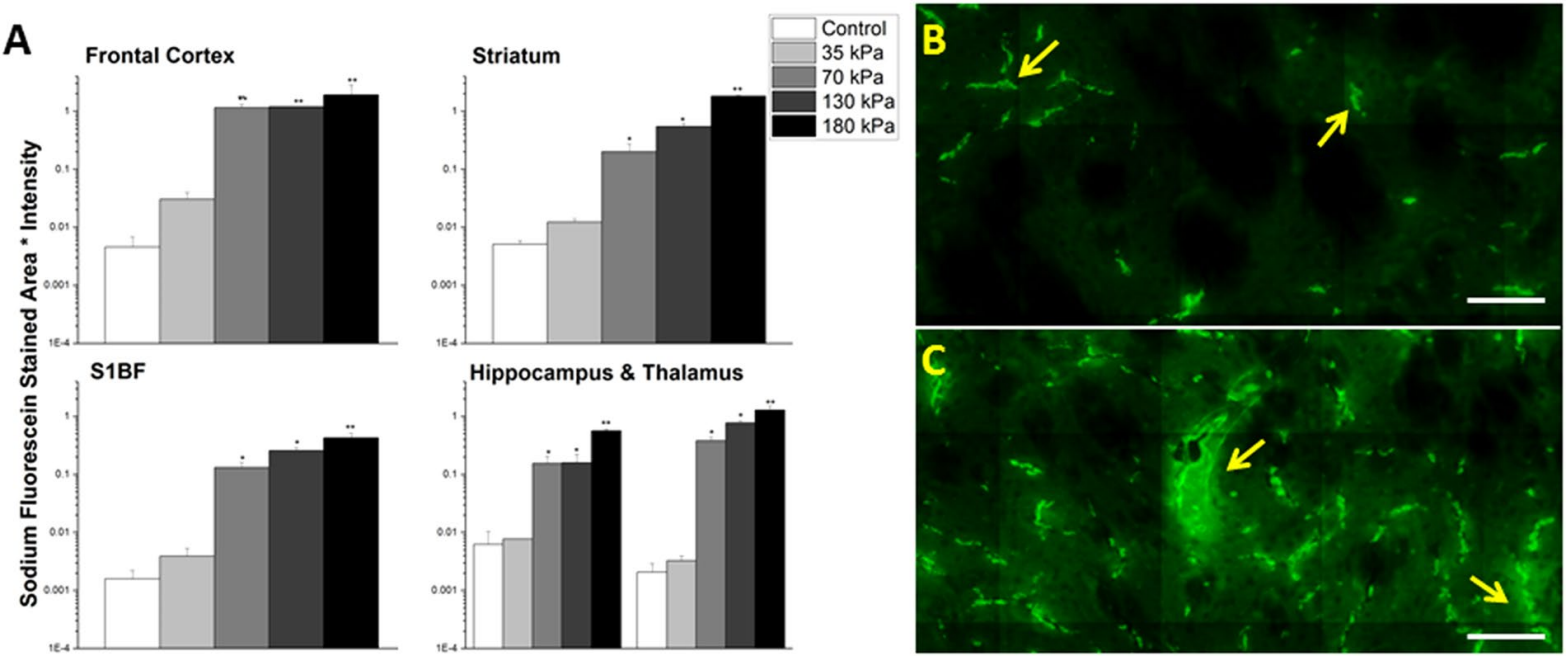
Quantitation of extravasation of sodium fluorescein for 35, 70, 130, and 180 kPa blast overpressures, 15 minutes post-exposure in frontal cortex, striatum, somatosensory barrel-field cortex, hippocampus, and thalamus using a semilog plot in order to capture magnitudinal differences (**A**). The striatum was chosen for illustrative purposes and to qualitatively depict the difference between 70 (**B**) and 130 kPa (**C**). Leaks appear longer and more intense with increasing overpressure in the same brain regions. *Indicates an intensity compared with control with a statistical significance of p < 0.05, **Indicates p < 0.01. Scale bar = 100 μm.

In order to determine the effects of blast overpressure on BBB permeability, groups of (n = 3) rats were exposed to mild shockwaves of 35, 70, and 130 kPa BOPs and sacrificed immediately after blast. While no measurable extravasation was induced at 35 kPa, there is clear evidence of leakage starting at 70 kPa (Figs 7 and 8) and extravasation quantitation for both tracers showed a tendency to increase with increasing overpressure. The fluorescence intensity increases over controls and were as follows for Evans blue at 35, 70 and 130 kPa, respectively as determined by post-ANOVA Tukey test: frontal cortex (0.014762, 6-fold, 0.826735, 340-fold, 0.898087, 370-fold), striatum (0.00983, 5-fold, 0.118309, 70-fold, 0.355441, 200-fold), somatosensory barrel-field cortex (0.0036, 1-fold, 0.101135, 30-fold, 0.215851, 70-fold), hippocampus (0.0.007685, 1-fold, 0.15515, 25-fold, 0.15437, 25-fold), and thalamus (0.003252, 2-fold, 0.380609, 180-fold, 0.773843, 380-fold) and for sodium fluorescein: frontal cortex (0.030414, 7-fold, 1.146571, 250-fold, 1.91895, 260-fold), striatum (0.012241, 2-fold, 0.201411, 40-fold, 0.545131, 100-fold), somatosensory barrel-field cortex (0.003914, 2-fold, 0.132123, 80-fold, 0.256239, 160-fold), hippocampus(0.013312, 1-fold, 0.172132, 20-fold, 0.205551, 25-fold), and thalamus (0.00404, 1-fold, 0.181371, 50-fold, 0.302787, 85-fold).

### Primary blast induces translocation of astrocytic marker s100-β into blood stream and monocyte infiltration into brain

**Figure 9:**
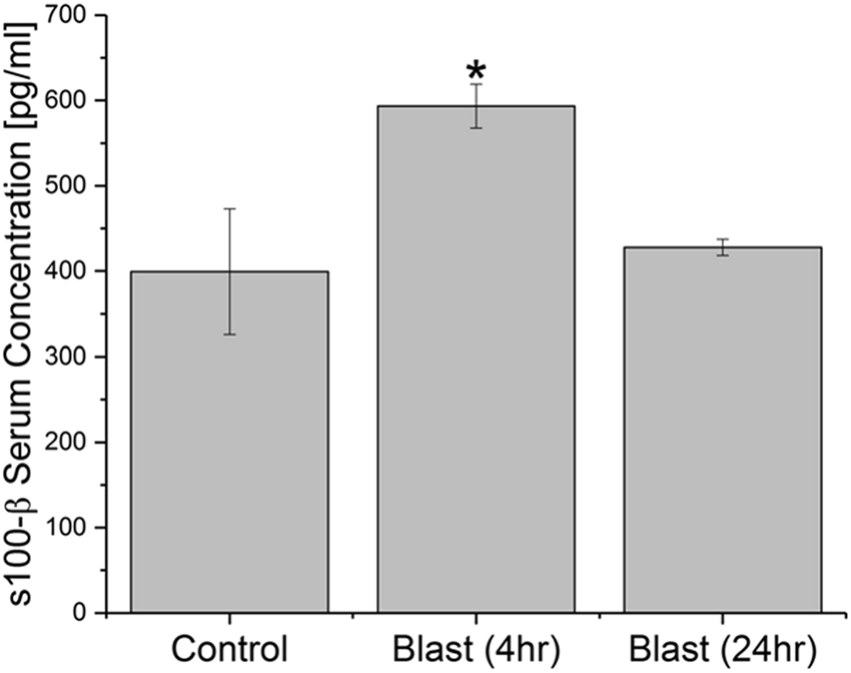
Concentration of s100-β in blood serum. Assay conducted for blast (180 kPa BOP) samples four and twenty four hours post-exposure and compared with controls. *Indicates a difference in intensity compared with control with a statistical significance of p < 0.05.

In order to more strongly display the presence of brain-specific proteins in circulation following blast injury, an ELISA was conducted for s100-β at 4 and 24 hours post-moderate blast (180 kPa) in serum samples (n = 3, Fig. 9). At four hours post-injury, concentration of s100-β rose from 399 pg/ml to 594 pg/ml, a statistically significant increase of 48.8% (post-ANOVA Tukey test, p = 0.037). However, after 24 hours, the protein levels in serum fall to 427 pg/ml, an increase of only 7% from control values (Tukey test, p > 0.05).

**Figure 10:**
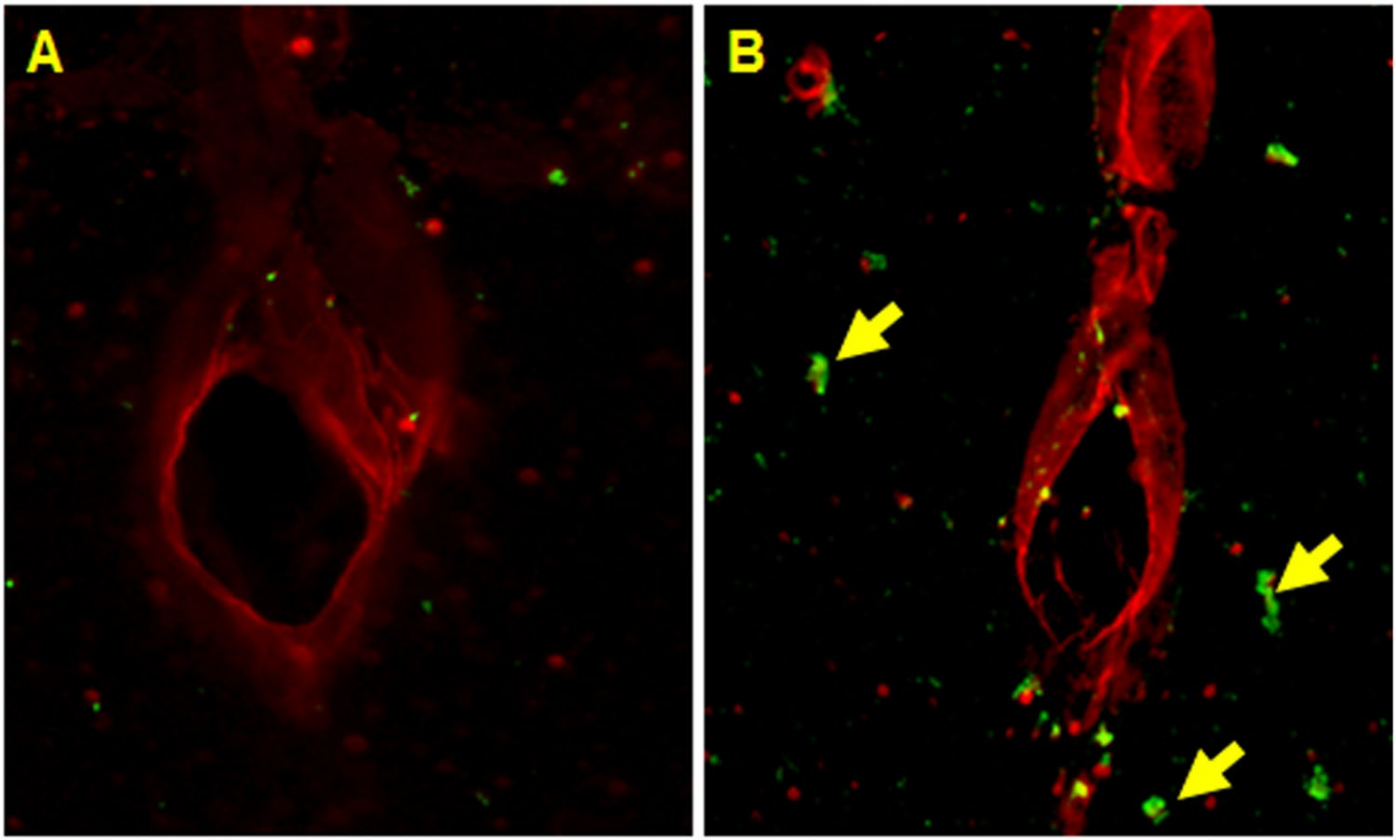
Double immunofluorescence of endothelial cell marker (RECA-1, red) and monocyte marker (CCL2, green) at four hours post-exposure in frontal cortex in animals from control (**A**) and moderate (180 kPa BOP) blast injury (**B**). Scale bar = 50 μm.

Similarly, as an alternative means to demonstrate the presence of blood-borne macromolecules/cells in the brain parenchyma, double immunofluorescence staining was performed for CCL2 (monocyte marker) and RECA-1 (endothelial cell marker) at 4 hours post-moderate blast in the frontal cortex (n = 3, Fig. 10). Results indicate that, qualitatively, the presence of monocytes around blood vessels increased following blast compared to controls.

## Discussion

This work focused on establishing spatial and temporal relationships of the BBB permeability as a function of overpressure in blast-induced traumatic brain injury. Through the use of two tracers (Evans blue and sodium fluorescein) injected intravenously in the lateral tail vein of the rat, the degree of the BBB disruption following injury was functionally assayed. At the sub-mild overpressure (35 kPa) very limited extravasation of dyes was observed. A breach in the BBB was first observed at a mild blast overpressure (70 kPa) which revealed a significant increase in barrier permeability almost immediately after blast (~15 min). In addition to extravasation of tracers (NaF and EB), absorption spectrophotometry was used to demonstrate the breakdown of the BBB by the presence of EB in brain parenchyma. The results of the absorption spectrophotometry offer interesting piece of corroboratory information; unlike gross blunt injuries, where there is a substantial amount of extravasated tracers recorded^59,60^, in this mild-moderate blast injury, a difference of only about 250 picograms of EB was observed between injured and control groups (Fig. 2).

It is interesting to note that the extravasation of the tracers showed a significant increase as early as 15 min following the blast, strongly supporting the hypothesis that direct biomechanical loading of the primary blast was able to disrupt the BBB (Figs 2 and 3). We should note that there is no physical impact of external objects such as in CCI or weight drop models, nor a specific fluid pressure in subdural space as in fluid percussion model. However, while the degree of BBB disruption appears large when compared to controls (which was negligible), the physiological as well as neurological state of the animal were not altered based on visual observation of the animal’s status including unaltered gait, righting and startle reflexes (data not shown). This strongly suggests that this injury can be classified as a subtle, tissue-level mechanical disruption of vasculature. Therefore, caution must be exercised when comparing these results with those from blunt injury models, where injury severity is greater and degree of extravasation is larger but the injury is highly local, mostly restricted to the site of external impact or fluid pressure^35,36,61^. As noted earlier, the degree of the BBB permeability increased further at 4 hours post-injury (Figs 4 and 5), which not only indicates the persistence of the BBB disruption but also indicates that, in addition to direct biomechanical loading (shock loads that lasts for a transient period of approximately 3 milliseconds), some secondary mechanisms may be activated post-injury and contribute to BBB disruption for hours after initial trauma.

While several secondary mechanisms have been implicated in the degradation of the BBB including oxidative stress^12,30,33,43^, matrix metalloproteinase activity and neuroinflammation^12,33,38,62^, pericyte detachment^15,63–65^, astrocytic end-feet swelling and detachment^66–69^, among others, precise involvement of one or more of these mechanisms contributing to BBB disruption in bTBI remains to be determined. Previous studies have cited maximum degree of damage to the BBB following mild-moderate TBI (although not blast) occurring between 4–6 hours of injury^8,35,39^. The lack of a detailed time course of BBB disruption in blast injury is a primary motivation for this work and a greater understanding of the temporal nature of the evolution of pathology and contributions of precise mechanisms during the time-course of BBB disruption in blast-induced neurotrauma will aid in diagnostic and therapeutic discoveries^70^.

The integrity of the tight junctions was also assessed in the current study immediately following moderate blast, four, and twenty-four hours post injury. Tight junctions are water-tight seals that connect adjacent endothelial cells across the brain vasculature^11,13^. These complexes are comprised of several proteins that anchor the tight junction to the surface of endothelial cells and represent a strong mechanical junction that is the foundation of the BBB. Several groups have studied the integrity of the tight junctions as a means to assess the state of the BBB^30,44,56,71,72^. In the present study, the abundance of tight junction proteins (occludin and claudin-5) was evaluated quantitatively via ELISA (Fig. 6A,B). In the acute phase of injury (~15 mins), a reduction in tight junction protein abundance was observed in both occludin (p = 0.012) and claudin-5 (p > 0.05). Although the reduction in claudin-5 was not statistically significant, it displays a strong tendency to decrease. These results strongly support our tenet that a direct mechanical loading may be able to break and dislodge tight junction complexes and cause subsequent reduction in integrity of the BBB, which may have manifested in the extravasation of the dyes. Further, at four hours post-blast, levels of occludin and claudin-5 were reduced (p = 0.002, p = 0.035, respectively) suggesting that, in addition to direct mechanical forces, secondary factors that are likely activated 4 hours post-injury also contribute to the reduction in TJPs. Such sustained decrease in these proteins may in part be responsible for greater compromise in BBB integrity 4 hours post-injury observed in the present study. While several factors have been implicated in the breakdown of tight junction proteins, oxidative stress and increased matrix metalloproteinase activity have been shown to contribute to the disruption of the BBB^12,30^. Moreover, restoration of the levels of occludin and claudin-5 observed in the present study within twenty-four hours post-blast corroborates the absence of extravasation seen at 24 hours post-blast.

While the reduction in the brain levels of tight junction proteins suggest a possibility of their dislodging by direct impact of the shockwaves, we sought to examine the accountability of these proteins in the blood stream, since it is likely that once dislodged, these proteins may be translocated into blood stream. Therefore, we estimated the levels of these proteins in serum samples by quantitative ELISA. Interestingly, we observed a close, inverse correlation on the levels of these proteins between brain homogenates and serum (Fig. 6C,D). Fifteen minutes post-injury, there was a significant increase in the amount of occludin (p = 0.012) and claudin-5 (p = 0.011) detected in the blood serum compared to controls. The amount of detected tight junction proteins further increased four hours post-injury (p < 0.001, p = 0.002) for occludin and claudin-5, respectively. These results not only corroborate the ELISA results in the brain tissue, but also give strong support to the assertion that the tight junction proteins are being mechanically dislodged from the tight junction complexes and being taken up into circulation. Moreover, a complete recovery of these TJPs to the control levels in brain and blood 24 hours post-injury corroborates well with the absence of any extravasation of EB or NaF which together indicate the possibility of resealing of the BBB at this time point.

Several studies report that increased levels of astrocytic protein s100-β in the blood indicate the breakdown of the BBB^12,30,40,73–75^. The current study shows increased s100-β in serum from animals exposed to blast provides additional support to our extravasation studies and together represent the involvement of astrocytic defects in the compromise of BBB following blast injury.

To demonstrate infiltration of any blood-borne cells entering the brain parenchyma, an immunofluorescence stain was performed to identify the presence of monocytes in the vicinity of vascular endothelial cells using CCL2 and RECA-1, respectively (n = 3, Fig. 10). CCL2 is a monocyte chemoattractant protein which presents on blood monocytes and is integral in monocyte mobilization while RECA-1 is a common vascular endothelial marker^76–78^. The increased number of CCL2 staining in the frontal cortex near blood vessels indicates that monocytes are leaking from the blood into the brain parenchyma four hours post-injury. This qualitatively supports the extravasation results for Evans blue and sodium fluorescein.

In the current study, differential degree of BBB permeability was observed spatially across different brain regions. Such differential blood-brain barrier permeability may, in part, be due to variations in the vascular architecture (density, size, orientation) in different brain regions. Cavaglia’s group characterized the variation in neurocapillary density in the adult rat hippocampus and cortical structures^79^. The hippocampal CA1 region revealed a significantly lower capillary density compared to CA3, but a much more extensive blood-brain barrier leakage. This may instinctively point to an inverse relationship between vascular density and BBB vulnerability; however Cavaglia’s studies also showed that neocortical regions have a much higher vascular density compared to neighboring gray/white matter junctions. Since gray matter regions (frontal cortex, thalamus, etc.) have a higher vascular density than the white matter regions, the mechanical shock loading in the acute phase of injury in conjunction with the onset of secondary mechanisms during latter stages may damage the brain regions containing higher vascular densities more than others. Supporting this tenet, our results and many other studies reveal higher degree of BBB damage in the frontal cortex (Figs 2 and 5)^34,43,58,72^. In addition to vascular density (number of vessels, vessel length, etc.), it is also possible that vascular orientation and cellular architecture may also be, in tandem, responsible for the observed spatial variation of BBB permeability. From a purely biomechanical perspective, a combination of vascular architecture and, to some extent, perivascular attachment to astrocytes may dictate the response to the sudden physical motion of capillaries leading to the mechanical disruption of tight junctions.

While it is strongly assumed that the propagation of shock uniformly travels and loads the whole brain, absence of BBB damage in cerebellum is interesting. The lack of functional BBB damage in the cerebellar regions has been reported by several groups, but there is still uncertainty to its cause^34,58,80^. Several groups have shown vascular volume in the cerebellum to be higher than that in the cerebrum^81–83^, but Holash et. al determined that this difference was due to the inclusion of pia vasculature^84^. Without pial vessels (which have a BBB quite different from the parenchymal vessels in terms of tight junction distribution and astrocytic ensheathment^85^), the vascular volume of the cerebellum and cerebrum are comparable. Most extravasation studies use intravascular tracers, which do not discriminate between pial and intraparenchymal vessels in the cerebellum. The authors speculate that the lack of extravasation in the cerebellum based on these methods is due to the presence of the pial vessels, but more work needs to be done to validate this hypothesis (ie. isolating parenchymal vessels).

Further, in the current study, based on the high magnification images (Figs 2, 3, 4, 5, 7 and 8) it is tempting to speculate that larger diameter blood vessels had greater leakage compared to smaller ones. Hypothetically, given a larger cross-sectional area, these vessels bear a greater brunt of the passing shock wave and hence are more damaged than their smaller counterparts. It is also possible that these larger vessels only appear to leak more because of a greater vascular volume: more vessel content, the more blood is able to leak out in the presence of a vascular rupture. In this case, vessels of all sizes would experience a similar mechanical load from the shock wave and show a similar pattern of leakage (with only differences in leakage volume, proportional to vessel size), which is consistent with the results of this study. When a shock/stress wave encounters vasculature, the biomechanical forces will be proportional to the projected area and the difference in acoustic impedance between the different materials that make up the local tissue construct. The resistance to deformation will be proportional to the structural integrity of the vasculature vis-à-vis the surrounding cellular architecture. As different subregions of the brain have different architectures and biomechanical characteristics, the forces, the deformation and hence the BBB leakage will be a function of the specific region under question even if the loading is identical.

After 24 hours following mild blast, a statistically significant decrease in extravasation was observed in all brain regions, which represents possible evidence of a BBB resealing in combination with the restoration of TJPs (Figs 4, 5, 6). A recent study also reported a reduction in BBB damage 24 hours after injury in mice exposed to moderate blast (100 kPa)^86^. This, however, may not preclude continued presence of phenotypic changes of the TBI in brain structures since vascular leakage of various blood born substances into the parenchyma may trigger secondary events such as microglial activation leading to neuroinflammation.

At all time-points, in all regions, the total amount of extravasated sodium fluorescein tracer was greater than Evans blue, an intuitive result given the difference in size between these two tracers. One needs to be careful in interpreting the results in terms of fold increase since the baseline data for the smaller molecule, sodium fluorescein, was higher than the larger Evans blue. In no region was there a collection of sodium fluorescein in the absence of Evans blue, meaning that in the current work, there was an insignificant number of breaches in the BBB which could accommodate the 376 Da sodium fluorescein but not the larger 69 kDa Evans blue.

The present study showing a strong tendency of higher extravasation in animals exposed to 130 kPa compared to 70 kPa in the acute phase of the injury suggests the BBB permeability changes are directly proportional to increasing BOP, at least in the acute phase of the injury. Interesting that 4 hours post-injury such magnitude of difference in the extravasation of EB and NaF as a function of BOP is absent. While the reason for this is not known, the authors speculate that differences in these injuries become more apparent after the acute phase of injury and such significant changes may be masked once secondary mechanisms begin to occur (4 hours), which may uniformly exacerbate injury conditions since, in the immediate phase of injury (~15 min), all leakage of the BBB is attributed to the mechanical insult of the shock wave. Therefore, the resulting biomechanical injury may not be grossly different between mild and moderate overpressures at time interval (4 h) where secondary mechanisms begin to occur. Indeed, such differential changes in BBB permeability have also been reported in other investigations^52,56^, which may superimpose the mechanical injury with the influences of secondary biochemical mechanisms (e.g., oxidative stress, neuroinflammation).

Our efforts to establish a sub-mild injury model of bTBI led to the identification of 35 kPa as a BOP that does not show any tracer extravasation. However, the BBB permeability changes are significant with a minimal BOP of 70 kPa and increased further as a function of increasing BOPs (Figs 7 and 8). These results indicate that the mechanical loading sustained at 35 kPa is insufficient to cause the significant damage to the BBB that was seen at 70 kPa and higher overpressures. Therefore, 70 kPa BOP offers a basal injury threshold for the BBB permeability changes to occur under primary blast loading. These studies together demonstrate that BBB permeability is a sensitive phenotypic marker for mTBI in the acute phase of the injury wherein a direct mechanical disruption is able to cause vascular rupture. Accordingly, alterations in the integrity of BBB may be considered a prognostic event to scale the injury severity (i.e., sub-mild to mild vs moderate TBI) as well as the extent of pathological outcomes in blast TBI.

In summary, this work addresses a clear gap in knowledge in the understanding of the BBB permeability in the pathophysiology of blast-induced traumatic brain injury. The results and conclusions presented herein should provide the baseline for future studies attempting to connect the BBB permeability and the pathophysiological progression of bTBI. However, while the authors maintain that these results are reproducible in age-matched rats exposed under the same loading conditions, it is important that the described experimental model is replicated as closely as possible in order to reproduce these results. For example, non-primary injuries will likely be observed if animals are located outside or near the end of the shock tube or if animals are not properly fixed and thus may reveal an altered injury profile.

## Materials and Methods

### Animal Preparation

A total of 88 adult, 10-week old male Sprague Dawley rats (Charles River Laboratories) weighing between 300–350 g were used throughout this study, in accordance with protocols approved by Rutgers University Institutional Animal Care and Use Committee (IACUC). Animals were housed at 22 °C with free access to food and water in a 12 hour dark-light cycle. Animals were divided among sham and injured groups for four different blast overpressures and three different time-points post-injury. All methods used throughout the study were performed in accordance with protocols, guidelines, and regulations approved by Rutgers University IACUC.

### Exposure to Blast and Tracer Injections

Rats were exposed to a single shock wave at the Center for Injury Biomechanics, Materials, and Medicine (New Jersey Institute of Technology) in the modular, field-validated shock tube described in previous publications^54–56,87^. Based on preliminary findings, we observed an obvious difference between sham and blast groups, where an n = 4 was sufficient to achieve a power value of 0.9 (α = 0.05 and combined SD of 0.819) based on power analysis. An n = 1–2 was added in case of mortality or inadequate perfusion. As we continued the study, we maintained an n = 5–6 for all blast groups (for 70 and 130 kPa groups, only three animals were used, but statistical significance was achieved). Prior to blast exposure, animals were anesthetized with 5% isoflurane, released in a chamber containing 95% air and 5% CO_2_, until unresponsive to noxious stimulation. At this point the rats were mounted and immobilized on a custom rat-holder in the test section of the shock tube. Sham animals were anesthetized and received noise exposure, but kept outside of the shock tube, away from the shock wave. Exposed animals were subjected to a single blast of 35, 70, 130, or 180 kPa peak overpressure and euthanized via transcardial perfusion-fixation at prescribed time-points (15 mins, 4 hrs, 24 hrs). Tracers were injected two hours prior to euthanasia (animals in the 15 min group received injections prior to blast exposure). Sodium fluorescein was administered via the lateral tail vein (376 Da, 20% dissolved in phosphate-buffered saline [PBS], 0.02 g/mL, 0.7 mL delivered) at the same time Evans blue was (69 kDa when bound to albumin, 2% solution dissolved in PBS, 0.002 g/ml, 0.7 mL delivered). Two hours were given as to give sufficient time for the tracers to circulate the body multiple times and perfuse even deeper neurovasculature across all experimental groups. Other groups have used both these dyes to assess BBB permeability up to four hours following tracer administration^34,88,89^. Half-life of EB in circulation was confirmed to be > four hours, while the same is true for NaF half-life^90–92^, instilling confidence that allowing two hours for circulation and extravasation for these tracers is adequate.

As a quality control measure, high-speed video was monitored and recorded with a Photron FASTCAM Mini UX100 operating at a framerate of 5000 fps to capture any substantial head/body movement during blast, in order to exclude the effects of secondary/tertiary injury from this study. Approximately two seconds of video footage were recorded per exposure and then saved via PFV (Photron FASTCAM Viewer) 3.3.5 software. Incident overpressure at the location of the animals in the test section of the shock tube was recorded at 1.0 MHz sampling frequency by a custom LabView program running on in-house built data acquisition system based on National Instruments PXI-6133 32 MS Memory S Series Multifunction DAQ Modules and PXIe-1082 PXI Express Chassis. PCB Piezotronics (Depew, NY) model 134A24 pressure sensors were used in all experiments.

### Tissue Preparation, Absorption Spectrophotometry, and *Ex-Vivo* Imaging and Analysis

Rats were perfusion-fixed two hours following tracer administration. Prior to perfusion, blood serum was extracted from the heart (left ventricle, approximately 3 ml volume). Rats were transcardially perfused with phosphate buffered saline (PBS) and brains fixed with 4% paraformaldehyde (PFA). Brains were then liberated from cranial vaults, immersed in 4% PFA for an additional 48 hours and cryoprotected through immersion in 30% sucrose. Appearance of brains can be seen in Fig. 1E, wherein the brains were completely devoid of blood in the vasculature, indicating a complete saline perfusion. Brains were then dissected into 100 micron sections using Rat Brain vibratome (Kent Scientific Corp.) and mounted on glass slides. Regions of interest included the frontal cortex, striatum, somatosensory barrel-field cortex, hippocampus, thalamus, and cerebellum. Each animal offered 30 sections across five regions that were analyzed. Slides contained between two to three sections, resulting in over 200 slides scanned throughout this study. Slides containing different brain regions were digitized (10x magnification) using Leica Aperio Versa 200 digital pathology grade slide scanner. Fluorescent intensities were quantified after excitation at 488 nm (sodium fluorescein), 50 ms exposure, and 594 nm (Evans blue), 125 ms exposure, using AreaQuant software specifically designed for this imaging application (Leica Biosystems) and expressed as average fluorescence intensity/unit area. This imaging technique allows for visualization of micro-structural details and digital scanning affords the ability to image large brain regions with no loss of resolution. In order to quantify fluorescence intensities, regions of interest were manually outlined in different brain section. For each channel (green 488 nm and red 594 nm), a minimum intensity threshold value was selected to exclude any background fluorescence from our calculation. The AreaQuant algorithm then determines if the intensity value of each pixel enclosed in the outlined region exceeds the minimum intensity threshold and outputs the total area of positive stain for each brain regions, the average intensity in each channel, and the expression profile of the tracers.

As a means to validate the results of fluorescent image quantitation, absorption spectrophotometry was conducted on homogenized frontal cortices extracted from control and acutely injured rats (n = 5, 180 kPa). Rats were sacrificed via saline perfusion (no fixation) and brains were extracted, sectioned, and frozen in dry ice. Absorption was measured and standard curve generated from seven gradient dilutions of Evans Blue. Experimental samples were plotted against the curve (R^2^ = 0.998) using SpectraMax i3 (Molecular Devices) microplate reader and SoftMax Pro 6.5 software. Output concentration was converted into micrograms per mg of brain tissue.

#### ELISA

As a means of alternative evidence for BBB disruption, tight junction protein changes were examined in the cerebral hemisphere by ELISA and immunoblot. Following perfusion with PBS, brains were excised from the skull and cerebrum was homogenized in CellLytic-M (Sigma) using sonicator with probe amplitude set to 45% on ice. Samples were then centrifuged at 14,000 g at 4 °C. The protein concentration in the samples was estimated by bicinochoninic acid (BCA) method (Thermo Scientific, Rockford, IL). Subsequently, samples were diluted in PBS and loaded onto ELISA plate (LSBio, Seattle, WA). Serum samples were also loaded onto same plate for tight junction protein quantification and separate serum ELISAs were run for s100-β. Plates were read in microplate reader (Spectra Max i3, Molecular Devices) at wavelength of 450 nm. All the Steps of ELISA procedure (washings, incubation time etc) were conducted in accordance with manufacturer instructions and samples plotted against a standard curve made up of eight samples (R^2^ = 0.995, 0.999 for occludin and claudin-5 respectively) using SoftMax Pro 6.5 software.

### Immunofluorescence

In order to further establish blood-to-brain leakage following blast injury, double-immunofluorescence studies for RECA-1 and CCL2 were performed in the frontal cortex, four hours post-injury (n = 3) as a means to detect infiltration of monocytes into the brain parenchyma. Following transcardial perfusion-fixation, tissue was cryoprotected in sucrose, and 20 μm thick sections were cut using Leica 1000 S vibratome. Sections were mounted on glass slides and washed with 10 mM PBS, fixed in ice-cold methanol (100%) solution for ten minutes at −20 °C. The tissue sections were blocked with 10% donkey serum at room temperature for 1 hour in PBS containing 0.03% Triton X-100. Fixed tissues were incubated overnight at 4 °C with respective primary antibodies to RECA-1 (Mouse monoclonal, Abcam, 1:50) and CCL2 (Rabbit polyclonal, Abcam, 1:50). Double immunofluorescence was performed using Alexafluor 594 for RECA-1 and Alexafluor 488 for CCL2. Slides containing different brain regions were digitized (20x magnification) using Leica Aperio Versa 200 fluorescent microscope and slide scanner.

### Statistical Analysis

Data are presented as mean + standard error of the mean. Statistical significance was determined using one-way analysis of variance (ANOVA) to compare mean fluorescence intensities of different brain regions for sodium fluorescein and Evans blue with a Tukey pairwise test done to determine differences between individual time-point and overpressure groups. Statistical comparisons were also made for each blast overpressure and each time-point post injury. Normalcy and population variance homogeneity were assessed with Shapiro-Wilk and Levene’s tests respectively. Differences between means were assessed and probability levels of p < 0.05 were considered statistically significant. Minitab 17 Statistical Software was used for all analyses and Origin 2017 was used for generation of bar plots. Bar plots presented are in semi-log scale in order to capture magnitudinal differences between groups. Fluorescent images were taken using Aperio Versa software and analysis and export done via ImageScope software (LEICA Corp.).
